# Student and resident perspectives on professionalism: beliefs, challenges, and suggested teaching strategies

**DOI:** 10.5116/ijme.5334.7c8d

**Published:** 2014-05-10

**Authors:** Abraham A. Salinas-Miranda, Emily J. Shaffer-Hudkins, Kathy L. Bradley-Klug, Alicia D.H. Monroe

**Affiliations:** 1 Community and Family Health Department, College of Public Health, University of South Florida, USA; 2 School of Psychology, College of Education, University of South Florida, USA; 3 Academic Affairs and Faculty Development, Baylor College of Medicine, USA

**Keywords:** Medical professionalism, focus groups, curriculum development, medical students, medical residents

## Abstract

**Objectives:**

The purpose of this study was to investigate the views of medical students and residents regarding the practice of professionalism, their perceived challenges, and ideas for the development of a new curriculum in medical professionalism.

**Methods:**

Data were collected from four focus groups comprised of 27 residents and medical students recruited from the University of South Florida Morsani School of Medicine and Residency Programs between January and March 2012. A questioning protocol was used to guide the focus group discussion. Data were transcribed for thematic analysis.

**Results:**

Learners expressed beliefs regarding key attributes of professional behaviors, factors perceived to be associated with lapses of professional behavior, skills that need to be taught, and strategies to teach professionalism from the learners’ perspective. Learners perceived that the values of professionalism are often disconnected from the reality evidenced in clinical training due to a myriad of personal and contextual challenges.

**Conclusions:**

Residents and students need help in negotiating some of the challenges to medical professionalism that are encountered in clinical settings. We recommend a learner’s centered model of curriculum development in medical professionalism that takes into consideration perceived challenges and strategies for modeling and reinforcing medical professionalism.

## Introduction

Medical professionalism is a “commitment to carrying out professional responsibilities, adherence to ethical principles, and sensitivity to a diverse patient population”.^1^ Such commitment is thought to be fueled by knowledge of a set of ethical principles and positive attitudes toward professional responsibilities.^1-3^ However, there is evidence of a widespread mismatch between the knowledge and attitudes reported by practicing physicians and their actual professional behaviors. This mismatch has been attributed to inconsistent or ambivalent learning models during training.^4^ Despite constant efforts of educational institutions and regulatory agencies in promoting medical professionalism training in the United States, the gap between the theory and actual behaviors remains unchanged.^5^ The acquisition of the behavioral skills of medical professionalism among medical students and residents depend on formal and informal educational experiences, as well as on a hidden curriculum.^6^ Formal education experiences include the intended and formally endorsed curriculum, which include explicit didactic experiences in the traditional sense. The informal curriculum includes informal unique experiences that occur during the interpersonal interactions in the academic center between students and faculty.^6^

Lastly, the “hidden curriculum” refers to a set of organizational structures and culture that influence how students learn how to be “doctors” in the real world.^6,7^ Thus, the hidden curriculum is represented by commonly held understandings or aspects of the daily clinical experiences that due to its implicit nature are taken-for-granted as behavioral norms.^6,7^ However, if the hidden curriculum is in contrast with the set of values transmitted in the formal curriculum, it becomes a set of obstacles or challenges that compete against professionalism goals during the training years.^7-9^ In this context, we argue that an improved understanding of the specific challenges related to medical professionalism experienced by residents and students can inform the development of strategies to mitigate the negative effects of the hidden curriculum, while enhancing the positive value of formal medical professionalism programs. A better understanding of the reinforcing mechanisms that influence the learning experiences and the socialization processes of professionalism is needed to inform the development of effective teaching strategies for medical school and residency programs.^10,11^ The purpose of this study was to ascertain the learners’ views regarding the practice of professionalism, their perceived challenges to engaging in professional behavior in practice, and to integrate their voices in the development of a new curriculum for medical students and residents.

## Methods

Four focus groups were conducted from January to March 2012 among medical residents and medical students from the Morsani College of Medicine at the University of South Florida. Approval was obtained from the university Institutional Review Board (IRB) prior to conducting the study. Given the study objective, we invited a purposive sample of medical residents and students who have participated in diverse clinical rotations from diverse hospitals of Tampa Bay to capture their insights on the practice of medical professionalism. Hence, Psychiatry, Internal Medicine, Emergency Medicine, and Pediatrics residents, as well as third year medical students, were invited to participate with an e-mail letter. A consent form describing the study, the benefits, and risks of participation was attached to an invitation letter. The informed consent form was also explained at the beginning of the focus group sessions, allowing for questions and clarification. Participation was voluntary and without monetary incentive. Focus groups were conducted in English by two trained facilitators external to the Morsani College of Medicine. To minimize moderator bias and method error, all focus groups were conducted by the same team of facilitators, who used the following questioning protocol:

1. When you hear the term professionalism, what comes to mind?
2. Think about some of your personal encounters with professional behavior. What are the best/worst examples that come to mind?
3. What are some of the best attributes and/or characteristics you heard related to professional behavior?
4. What do you think are some of the major factors that were given in the examples that showed a lapse of professional behavior?
5. What makes it difficult for medical students or residents to be “their professional best” in situations like the ones you just described?
6. If you had a chance to give advice to a team who is developing a curriculum focusing on training professional behavior, what kinds of skills or issues would you tell them to include?
7. What do you think would be the best way for students/residents to learn a topic like this?

Each focus group was approximately one hour in duration and held at a time of convenience in a designated private conference room at local hospitals or at the university. Each focus group was audio-recorded and subsequently transcribed by a professional transcription company. However, we also confirmed the transcription by playing the audio file of illustrative quotes for accuracy. Names were omitted in the transcriptions to assure confidentiality. Three qualitative data analysts conducted thematic analyses of transcriptions and field notes, which included the two focus group facilitators (KB, ES) and an independent data analyst (AS). Textual data were managed with the software MAXQDA, Version 2007.^12^

The analysis consisted of a three step approach to coding: open coding, axial coding, and selective coding. During open coding, analysts independently read through the focus group transcripts several times and applied tentative labels to textual data (i.e., examples of participants’ words) that summarized key points or answers to the focus group questions. Memos were noted with the properties of each code. At this step, we conducted an intercoder reliability analysis to assess the independent assessment of transcripts by the coders and comparing the agreement between the coders.^13,14^ This assessment was conducted with direct data and at surface-level.^15^ Particularly, we computed the average pairwise Cohen’s Kappa statistics using the software Recal3.^16^ This analysis demonstrated substantial agreement between the coders (Coders 1 & 3 = 0.786, Coders 1 & 2 = 0.817, Coders 2 & 3 = 0.751), which supported the dependability of the open coding.^17^

Next, axial or thematic coding was conducted. For this purpose, an analysts meeting was convened to identify and discuss relationships among the open codes, which resulted in a set of thematic categories. For selective coding, each analyst reread the transcripts and selectively coded any textual data that related to the thematic categories identified. A final meeting was convened to discuss areas of considerable disagreement and to resolve the found differences. Illustrative quotes were selected. Exact quotes were put in double quotations marks, while paraphrased statements were indicated by single quotation marks.

### Focus groups findings

Three focus groups were conducted with medical residents and one with medical students. Most participants were medical residents, female, and less than 30 years of age (Table 1).

A total of 106 pieces of textual data across focus groups were coded, which were grouped into the following emerging themes: examples of professional behaviors, key attributes of professionalism, personal and environmental challenges, essential skills, and ways to teach professionalism.

### Examples of Professional Behaviors

Conceptual notions on professionalism were mentioned across focus groups, including: respect, integrity, honesty, courtesy, politeness, caring, appropriate interactions, acting as a team player, knowledgeable in the medical science, and punctuality. Most notably, participants identified several examples of professional and unprofessional behaviors. The most common examples of professional behavior emphasized the importance of putting the patient interest first, as summarized by one resident:

“Being able to treat the patient, give the patient the best care possible as far as the patient and the family being angry and not have that affect the patient’s care.” *(Female, resident)*

**Table 1:** Characteristics of participants (N=27)

Variable		Frequency
Participants per group		
	Student group #1	7
	Resident group #2	11
	Resident group #1	6
	Resident group #2	3
Gender		
	Female	21
	Male	6
Age (in years)		
	25-27	10
	28-30	10
	31-33	5
	>34	2
Medical students (3^rd^ year)		7
Specialty to pursue		
	Internal Medicine	4
	Surgery	1
	Obstetrics/Gynecology	1
	Emergency Medicine	1
Medical residents		20
Year in Residency		
	1^st^ year	5
	2^nd^ year	3
	3^rd^ year	8
	>4^th^year	4
Specialty		
	Pediatrics	11
	Psychiatry	6
	Internal Medicines/Pediatrics	2
	Emergency Medicine	1

Other salient examples of professional behavior were noted in situations where the doctor was being respectful and appreciative of staff members and patients,

“I had two ‘attendings’ that were just excellent role models for professionalism. At all hours of the day, even late at night, even late on shifts, always respectful, thorough and detailed in their treatment of patients and families. Great examples.” *(Male, resident)*

A related example was teamwork where the clinicians were willing to help out others and facilitate the daily work of the healthcare team:

*“rounding up the team, everybody, daily rounds, and teamwork”.* (Female, medical resident)

Participants also noted that,

“seeing attendings who can, in a constructive manner, give you feedback – either positive or negative feedback – can actually enhance your learning.” *(Female, resident)*

Recognition of their own mistakes to patients and apologizing accordingly was also noted as an example of professionalism:

“I see a lot of people and they think they made a mistake, they go in and talk to the family and saying - I apologize for this happening - but let them know this is what’s happened. Mistakes happen to people.” *(Female, resident)*

Other examples included maintaining adequate interactions with patients, students, residents, nurses, and other staff members, as well as following rules of punctuality, and attire. Recollections of unprofessional behaviors were also noted. Situations where health care professionals were “not taking responsibility for decisions they’ve made and blaming it on somebody else” (Female, resident) were noted as negative behaviors that can affect the management of the patient. Another example of unprofessional behavior occurred where a clinician, a team of clinicians, failed to conduct a thorough assessment of the patient, compromising the quality of care:

“…when I was in medical school, I had a woman in labor and my resident would not give her pain medication because she was a junkie. It’s the only time I ever got into a shouting match with a resident…So yeah, she’s a junkie, but she’s also in pain. Our job’s not to punish her. It’s to care for her. To me that’s unprofessional.” *(Male, resident)*

Treating others harshly or disrespectfully were unprofessional behaviors highlighted, when,

“a particular attending started yelling at one of the colleagues, another junior resident, in front of other health professionals” or “…negative and derogatory comments about the family members or the families.” *(Female, resident)*

Such inadequate forms of communication could occur between nurses and students, questioning students in front of others, and even with derogatory comments about each other. Less frequently mentioned examples were senior healthcare staff treating “students like they are there to work only with no educational value” (Female, student). One student noted that failure to protect patient confidentiality is also a concern coming from residents:

“In the ER, there was a resident that was coming in and out of one of the rooms, and he kept saying, “I’m going to go do a pelvic, and now I need somebody to sit in with me…and he kept saying it over and over while he was walking around the hallway.” *(Female, student)*

Another participant referred to failures to broach sensitive issues with patients and staff, suggesting that more adequate forms of open communication should be developed instead:

*“*failing to broach sensitive topics of conversation with other patients or colleagues. Certain things, like the sexual history, often fail to be elicited because of the level of discomfort, but as professionals we need to overcome that. It is often a very integral in the patient’s life.*”* *(Female, medical resident)*

### Key attributes of professional behavior

Key attributes or characteristics of medical professionalism identified by learners were effective communication, respect for others, approachability, and being thorough:

“Good communication…along the same lines of communicating well, good documentation. You can communicate but if it’s not written down, and the night team comes on and they don’t have it in writing they might not know what your plan is.” *(Female, resident)*

“Being not just respectful to patients but also to their colleagues, amongst colleagues.” *(Female, resident)*

“Having a good attitude, being approachable, and patience with your coworkers.” *(Female, resident)*

Other examples included professional behaviors beyond the work setting as to:

“Being the same person in front of family as you are whenever you come out, just being as good in real life as you present yourself.” *(Female, resident)*

and recognizing one’s own limitations,

“Know where your troubled spots are and asking for help.” *(Female, medical resident)*

Also a key attribute was displaying compassion and empathy,

“Because of the nature of what we do, and with health insurance coverage, the time that we get to spend with our patients is not much time, but still, a look, pat them on the shoulder; you don’t have to be so cold about it.” *(Female, resident)*

Being a team player (e.g. “…working well with your colleagues, really trying to help everyone else to get everyone's work done” was also recognized as a key characteristic.

### Challenges for doctors-in-training to be “Their Professional Best”

Participants recognized that some of the obstacles to implementing professional behaviors arise from personal challenges, such as stress from personal life that affects interactions in the workplace. For example, one participant noted:

“Sometimes your personal life incurs on your professional life and it ends up affecting your professional life without realizing it.”*(Female, resident)*

Not taking time to reflect is another personal factor:

“We don’t really take time to feel what we’re feeling or identify what’s going on with us. If we’re getting frustrated with a patient and you continue to argue and it escalates into something that people can say that’s obviously not appropriate. In that case, you could identify and say: this is not headed down a constructive direction.” *(Male, medical resident)*

Another personal issue was a lack of experience in dealing with emotionally distressed patients:

“It’s a lack of experience in dealing with patients that are often whiney, demanding, entitled, and unpleasant. Sometimes they’re not nice people, but it’s frequently because they’re hurting. We’re not seeing them at their best, and they’re scared.” *(Male, resident)*

Being overly tired or fatigued as a result of clinical work and performance at work and becoming routine were other challenges faced by learners:

“…when we’re overworked, it can turn into a job, and you’re like – oh my gosh, I’ve got to get through this number of patients today.” *(Male, medical resident)*

Lack of personal interest in certain rotations (lack of motivation for certain specialties), and not knowing roles and responsibilities during clinical rotations were also noted. Participants also highlighted environmental obstacles that hindered professionalism. One notable external factor was the presence of negative role models that sometimes displayed unprofessional attributes, in particular,

“unprofessional behavior coming from other staff or other medical professional because it's easier to sort of fall into that reaction after coming at you.” *(Male, medical students)*

Negative role models were typically seasoned physicians and residents whose actions discouraged students to use alternative ways to interact or communicate. One participant noted:

“If you don’t work with professional residents or professional attendings, you won’t be a professional medical student.” *(Male, resident)*

Conflicts with nurses were noted by medical students and residents as a barrier to acting as their professional best, particularly in regard to sharing procedures (i.e. turf issues). Examples of lack of respect toward new students or residents included:

“…lack of respect within the nursing staff. Coming in as a new doctor and still being in the training process, and they don’t treat you very nicely. So as nice as you are in the beginning, that attitude starts reflecting back.” *(Female, resident)*

“…medical students don’t feel appreciated or they are contributing.” *(Female, resident)*

Another external factor was the lack of constructive feedback regarding key professional attributes, which could have been used as reinforcing mechanism to the students or residents from those higher in the hierarchy (e.g. attending, other residents, or nurses) but instead represent a missed learning opportunity. One participant noted:

“Nobody grades you on whether or not you were respectful or you cared about them; or if you took the extra time; or if you patted them on the shoulder, or you listened to them talk about their problems.” *(Female, resident)*

“Certain services that have the tendency to have you do a lot of work without teaching anything. So it’s not motivating to you to want to do the work, and someone’s giving you what it should be a reciprocal process, so there’s a lot of frustration.” *(Female, resident)*

Other external factors included job-related aspects such as paperwork or administrative workload,

*“*…just getting through the bureaucracy of stuff, the paperwork, or you have to call this person to get this done, or you didn’t fill this form out, and …you don’t even want to have to do it because you have to deal with so much stuff just be able to treat the patient*”*- *(Female, resident)*

Time constraints related to shift change (‘in the rush of shift change following service, things get kind of hectic.’-Female, student), long working hours (‘30 hour shifts’- Female, resident), and other logistical barriers (‘Maintain patient confidentiality is hard to do at times because of the ER patient load. There’re two patients per room!’- Male, resident)

Conflict between the goals of professionalism and other performance measures was also perceived, such as when the organizational goal was to have fewest inpatient days and writing descriptive notes vs. acting on the best interest of the patients (what patients wanted). This aspect was explained by one participant as follows:

“If you have an hour, you can spend 10 minutes talking with the patient and 50 minutes writing the note and ordering everything…or you can spend 50 minutes with the patient, and then scramble in 10 minutes and do a horrible note, and people will assume you’re doing a bad job. When really, in that situation, you’re doing the best by the patient. And those are the patients who have the best satisfaction. So, those are kind of conflicting pressures.” *(Male, resident)*

### Teaching points: essential skills and ways to teach professionalism

Participants identified the gap in skills and key teaching points for the development of a curriculum in professionalism. Effective communication was a highly regarded skill overwhelmingly noted. Examples included: showing mutual respect and reciprocity when communicating with patients, using assertive communication between the clinical hierarchies and with peers, appropriate ways to complain or voice concerns when conflicts occur, effective communication to ensure positive interactions with patients’ relatives and different levels of staff. One participant summarized:

“You want to be taught how to communicate knowledge in a non-condescending fashion when you’re dealing with colleagues and residents, you want to be able to just communicate back and forth and have a nice flow of information on the same level. You must be able to listen. That’s almost critical to the position” *(Male, resident)*

Another important issue to be addressed is the establishment of clear roles and expectations in the diverse settings. This included setting demarcated roles for students, nurses, and residents early in the clerkship and receiving continuous constructive feedback on role performance. One participant echoed:

“If I would have known that I’m supposed to be doing that, I would do it. Why did you wait until two weeks into the service to tell me: by the way, you haven’t been doing this” *(Female, resident)*

Another need was to learn ways to effectively deal with “difficult patients”; for example, in situations where the patients were acting scared or upset and there was a need to reassure them adequately. Participants also recognized the need for an improved understanding of patient’s views and the need to find ways to provide reassurance and empathy to patients. One participant suggested:

“The little trick I use is I pretend, especially with unpleasant patients, I pretend I’m related to them. Pretend it’s your aunt, uncle, and what would I do for them? That’s what I need to do for this patient.” *(Male, resident)*

Others noted the need for demonstrating respect for all other professionals, develop skills in coordinating teams, how to best maintain patient confidentiality, and time management. Regarding strategies to teach professionalism, participants recommended the implementation of group orientations by an effective communicator during the clerkships and service rotations in order to improve the shared understanding of not only the rules and responsibilities for students but also for the other members of the medical team:

“…having a sit down or forum, or have somebody from each discipline come in like in the circle and you just talk about what you do, what your role is, what you think your job is, and then what you think the other people’s jobs are and then you can correct each other, or enlighten people as to what your role actually is.” *(Female, resident)*

Participants indicated that all team members play a key role in the education of medical doctors and that they need to have a general understanding of all relevant clinical activities beyond the required procedures. An important suggested strategy to learn professional behaviors was exposures to positive role models and the need to model behaviors for students and residents by experienced and successful medical professionals. It was evident that participants wanted ongoing feedback to be more structured or scheduled as it is often something that gets forgotten or falls to the wayside. Feedback should be guided by a trusted and respected mentor or role model, which could provide not just an opportunity for realizing one’s own deficiencies, but also being mindful about the issues faced on a day-to-day basis. For example, one resident noted that,

“seeing attendings who can, in a constructive manner, give you feedback, for positive or negative feedback, actually enhances your learning and feel that respect from a colleague who is superior to you or someone’s who should be a role model for you.” (Female, resident)

Another participant echoed

*“I honestly feel like the best thing that I would want is someone to come tell me like this is what it's like.”* (Female, resident)

Experiential learning, or learning by doing, was highly regarded as a way to observe, model, and reinforce professional behaviors. In particular, participants suggested practical case scenarios where students can have the opportunity to face a difficult situation, identify best and poor examples of medical behavior, and discuss alternative ways to address the situation while maintaining professional attributes. One participant stated:

*“We can all probably agree that a lecture or an internet module or something like that isn’t really an effective way. It is learn by doing.”* (Male, resident)

Participants indicated the potential use of role playing (*“A situation where people go through that process where people are upset, and learning how to talk through it without getting upset yourself”*-Female, student), consisting of students taking different roles within the clinical hierarchy and attempting to resolve a situation in the best interest of the team *(e.g. ‘Set up the different scenarios that people could have happened’-Female, resident).*

These strategies could be efficiently supplemented with discussion forums as an opportunity to not only share experiences and concerns. One resident noted:

*“we can have a resident and an attending in the room with us, and they each tell us from their perspective their difficulty with dealing with it. Just being able to talk in a round table type discussion with a very small group”* (Female, resident)

While participants recognize a place for formal classes, they noted that classroom-based lessons often have felt contrived or not sufficient for changing one’s professional behavior, as pointed by one participant:

*“Class to teach professionalism and tell people to have a positive attitude. But it is not like everybody's going to have a positive attitude because it is also their personality.”* (Female, resident)

They noted that assessment of professionalism must be conducted continuously, starting as early as the admission to medical school:

*“I wish there was a better way to screen the people that they hire, the people that we let into medical school”* (Female, medical resident)

In this context, some participants highlighted the perception that professionalism is more about internal factors or inherent traits, and thus emphasized the importance of including professionalism as a factor within medical school selection criteria. Other complementary ways to reinforce professionalism included the use of self-reflection / mindfulness. One participant reflected upon her own situation:

*“It’s okay to have unprofessional feelings, but it’s how you act on that feeling that defines a professional.”* (Female, resident)

Reminders or visual cues *(“a little card, a print out, a little reminding card for students. Something to make it personalized just to get us to remember….”-*Female, resident), and the use of a buddy system to keep each other in check were also mentioned. The above themes and examples are depicted in Table 2.

**Table 2:** Focus group themes

Main theme	Sub-theme and examples
Reported behaviors displayed by role models	Best examples of medical behaviors:
	Patient-centered care, being respectful and polite, teamwork, recognizes mistakes and apologize, teacher-learner exchanges, adequate interactions, and following conduct rules.
	Worst examples of medical behaviors:
	Gap of ownership and responsibility, not being thorough, mistreating others, inadequate comments, just work with no educational value, failure to broach sensitive issues, and failure to protect confidentiality
Key attributes	Essential attributes of professional behaviors:
	Effective communication, respect for all persons, approachable, being thorough and detailed in patient care, authenticity beyond work setting, compassionate care, recognize your troubled spots, team player, confidentiality, and integrity of actions.
Challenges for medical professionalism in practice learning settings	Personal factors:
	Personal stressors, overly tired, inexperience in dealing with emotionally distressed patients, falling into a routine, unmotivated for clinical rotation, undefined roles, and not taking time to reflect
	Environment factors:
	Negative role models, lack of appreciation, lack of constructive feedback, a lot of work without teaching, shift changes, long working hours, paperwork/ administrative load, conflicting pressures, and logistical barriers.
Teaching points for curriculum developers	Skills that need to be addressed in medical education from the perspective of learners:
	Communication skills training, setting clear roles and expectations, improved understanding of the patients’ views, dealing with difficult situations or persons, time management, how to maintain patient confidentiality in the rush, and respect for other professionals.
	Teaching strategies:
	Discussing roles, role modeling, experiential learning, practical scenarios, role playing, assessment of professionalism behaviors, discussion forum or round table, formal class, buddy system (peer-to-peer system), reminders/ visual cues, and self-reflection/ mindfulness

## Conclusions

Our study was a qualitative assessment of perspectives of medical residents and students regarding the practice of professionalism, their perceived challenges, and ideas for the development of a new curriculum in medical professionalism. Learners were able to identify preconceived notions of professionalism that were consistent with the literature on medical professionalism, indicating that they held adequate knowledge regarding the value of medical professionalism. For example, learners expressed beliefs regarding key attributes of professional behaviors that were similar to the findings from previous studies such as those pertaining to patient treatment decisions, communication, professional duties, and quality of care.^18^ However, it was clear that learners perceived that the ethical values of professionalism were often disconnected from the reality evidenced in clinical training. In this regard, participants noted several personal and contextual challenges to professionalism, which they associated to lapses of professional behaviors. Previous studies have reported that students suffer experiences of “powerlessness” and conflict between what they have learned in early years of medical education and what they see role modeled at the hospital.^19^ This mismatch between perceived values and reality was also evident in our focus groups. Such tension between knowledge and practice suggests that besides formal training in the conceptual aspects of professionalism, residents and students also need help in negotiating some of the challenges to medical professionalism that are encountered in clinical settings.

Several challenges were identified in this study, which have been scarcely discussed in the literature. Conflicting pressures from the organizational environment, how negative role models affect the students’ behaviors, and lack of constructive feedback are just emerging themes identified in this study. Although we recognize that changing the organizational environment of hospitals could be a daunting task, we consider that it should be part of the agenda of medical education institutions to maintain system-wide mechanisms that promote professionalism among members of the clinical team and in the encounter with the patients and their families. Furthermore, the real world will present with unpredictable and stressful situations for which the learners need to be ready to react professionally. In this context, medical educators and students need to devise strategies to handle difficult situations in a successful manner without jeopardizing their professional integrity.

More effective curriculum development on medical professionalism is needed. Several didactic forms of instruction on the topic of professionalism have been recommended.^20^ However, our study indicates that implicit forms of instruction continue to be the main source of learning professional behaviors. For instance, the students’ comments about positive and negative role models denote the importance of the so-called “hidden curriculum”.^21, 22^

Indeed, many of the examples noted by focus group participants highlighted the importance of role modeling and observational learning, which has also been noted by other authors.^23-25^ Such situation suggest a potential application of social learning theory for the development of curricula in professionalism.^26, 27^

In this study, participants suggested several strategies to enhance professionalism skills that attend to the personal challenges and contextual factors that affect residents and students. Notably, beyond day-to-day observation of mentors, students indicated that a more structured process to receiving feedback on their own professional behavior would be helpful in setting specific goals for improvement for which positive role models play a critical function. Thus, we recommend that medical educators promote learner-centered strategies to assist future medical professionals in negotiating common challenges related to medical professionalism.^28^

Results of the current study should be considered in the context of certain methodological limitations pertinent to qualitative research. First, although a purposive sample was utilized in the current study, participant groups were biased toward gender and student status (i.e., more females than males, and more residents than medical students). Hence, the perspective of males and students was underrepresented and require expansion in future studies. Also, due to the limited number of focus groups, it is likely that theoretical saturation was not reached and we recommend the replication of this study with other learners’ groups. Inherent to the type of research, generalizability to the larger population of medical students and residents is limited. However, our findings were authentic and credible, as they were based on the direct words and opinions of medical residents and students. Furthermore, although our study findings are transferable to similar groups of medical residents, the particular views of some groups of residents (e.g. surgical specialties) and students (4^th^ year students and interns) may have not been represented in the focus groups. Future research should explore the perspectives of learned professionalism among those underrepresented in this study to determine whether findings are similar. Despite the noted limitations, this study expanded our understanding of the authentic challenges faced by students and residents on professionalism with regards to personal and environmental factors that influence the acquisition of professional behavior attributes, and the potential ways to overcome them.
